# Assembly and analysis of the complete mitochondrial genome of endangered plant *Tilia amurensis* Rupr

**DOI:** 10.3389/fpls.2025.1686403

**Published:** 2025-12-03

**Authors:** Xiao Liu, Furong Lin, Shasha Zhai, Zhicheng Wu, Lei Gao, Nana Sun, Wenchao Yan, Jitong Chen, Dan Liu

**Affiliations:** 1 Qufu Normal University, Jining, China; 2 State Key Laboratory of Tree Genetics and Breeding, Key Laboratory of Tree Breeding and Cultivation of State Forestry and Grassland Administration, Research Institute of Forestry, Chinese Academy of Forestry, Beijing, China; 3 Shandong Provincial Center of Forest and Grass Germplasm Resources, Jinan, China; 4 Shandong Normal University, Jinan, China; 5 Hebei Normal University of Science and Technology, Qinhuangdao, China

**Keywords:** *Tilia amurensis* Rupr., endangered plant, mitochondrial genome, repetitive sequence, chloroplast-derived sequence

## Abstract

**Introduction:**

*Tilia amurensis* Rupr., a deciduous tree of the genus *Tilia* in the Malvaceae family, is classified as a second-class key protected wild plant in China. This study aimed to sequence and analyze the complete mitochondrial genome of *T. amurensis* to (i) aid the understanding of phylogenetic relationships within the Malvaceae family, (ii) clarify the taxonomic status of genus *Tilia*, and (iii) provide a molecular basis for the conservation and genetic breeding of this endangered species.

**Methods:**

*T. amurensis* mitochondrial genome was sequenced and assembled using the Illumina and Oxford Nanopore Technologies platforms. Additionally, bioinformatics analyses were conducted, along with gene annotation and functional analysis, to identify repeat sequences and chloroplast-derived fragments.

**Results:**

The assembled mitochondrial genome of *T. amurensis* was found to be a single circular molecule with a total length of 830,088 bp and a GC content of 44.97%. A total of 61 genes were annotated, including 20 tRNA, 3 rRNA, and 38 protein-coding genes. Moreover, 472 RNA editing sites were identified among the protein-coding genes. We also detected a high abundance of repetitive sequences and chloroplast-derived fragments. Specifically, the genome included 264 simple sequence repeats, 19 tandem repeats, and 855 pairs of scattered repeats, predominantly comprising short fragment repetitions. Furthermore, 22 chloroplast homologous fragments (with a cumulative length of 8,138 bp) were identified, accounting for 0.98% of the total mitochondrial genome length. Phylogenetic analysis based on the mitochondrial genomes of 35 plant species confirmed that *T. amurensis*, within the Malvaceae family of the Malvales order, is consistent with the latest classification proposed by the Angiosperm Phylogeny Group.

**Discussion:**

This study reports, for the first time, the complete mitochondrial genome sequence of *T. amurensis*, revealing its high reproducibility and notable chloroplast-to-mitochondria DNA transfer. These findings provide valuable data for understanding the evolutionary dynamics of the Malvaceae mitochondrial genome. Furthermore, they establish a molecular foundation for future research on the endangerment mechanisms of *T. amurensis*, its genetic diversity, and development of effective conservation strategies.

## 1 Introduction

*Tilia amurensis* Rupr., belonging to the genus *Tilia* of the Malvaceae family, is a deciduous tree species. It is classified as a nationally endangered and second-class key protected wild plant in China (The Wildlife Protection Department of the National Forestry and Grassland Administration, 2021). A valuable broad-leaved tree species, *Tilia amurensis*, commonly grows at altitudes ranging 600–900 m and is primarily distributed in the mixed forests of Heilongjiang, Jilin, and Liaoning provinces in Northeast China (Wang, 2023). There are also some scattered areas in Beijing, Hebei, and Shandong (Gao and Liu, 2025). *T. amurensis* is characterized by a large crown and an elegant shape, making it suitable as a street tree and an excellent species for landscaping. Owing to its fine and elastic wood, it is an excellent raw material for construction work (Qi, 2004). Additionally, it is recognized as an important nectar plant with economic value (An, 2023). *T. amurensis* requires highly fertile soil and prefers sunlight, but shows moderate tolerance for shade (Zhang et al., 2024). In its native habitat, the reproductive rate of this species is high, whereas its proliferation rate is low (An, 2023). Wild *T. amurensis* exhibits seed dormancy, which hampers population renewal and contributes to its declining state (Chun et al., 2022). The combined effects of natural conditions and human activities have led to a reduction in large-diameter individuals in natural forests, placing the species at increasing risk of endangerment.

Both domestically and internationally, *T. amurensis* research has primarily focused on propagation techniques such as tender branch cutting and seedling trait studies (Kong et al., 2020; Yang et al., 2024). Other studies have focused on seedling growth and cultivation technology (Yu et al., 2023; Fu et al., 2023; Luo, 2025), community niche characteristics and interspecific relationships (Bai and Shi, 2023; Guo et al., 2024), as well as growth and physiological traits (Jiang et al., 2025; Zhang et al., 2024; Zhang and Fan, 2024). Additional topics include leaf decomposition rates (Xu et al., 1998) and genetic diversity assessments (Gao and Liu, 2025). Genetic research on *T. amurensis* mainly includes comparative transcriptome analyses during somatic embryogenesis, which have identified potential signaling pathways involved in embryogenic development (Kang et al., 2021). Moreover, next-generation sequencing technology has been employed for the development and identification of new microsatellite markers of *T. amurensis*, providing a basis for further research on genetic diversity and conservation of natural populations (Chun et al., 2022). Comparative chloroplast genome analysis of *T. amurensis* has identified the highly variable regions useful for phylogenetic research and evolutionary classification (Yan et al., 2022). Furthermore, genetic diversity analyses using simple sequence repeat (SSR) markers from 16 germplasm origins have been conducted, allowing the construction of fingerprint maps to guide germplasm preservation (Gao and Liu, 2025).

To date, the mitochondrial genome of *Tilia* (including *T. amurensis*) remains uncharacterized. In particular, studies focusing on the structural characteristics of the *T. amurensis* mitochondrial genome are essential to clarify its phylogenetic status within the genus *Tilia* and to elucidate its evolutionary mechanisms. The present study aims to address this gap by sequencing, assembling, annotating, and analyzing the complete mitochondrial genome of this endangered and protected species. The key objective is to provide molecular evidence supporting phylogenetic research on *Tilia* and the Malvaceae family, and to establish a foundation for the conservation and sustainable utilization of *T. amurensis* genetic resources.

## 2 Materials and methods

### 2.1 Experimental materials and sequencing

The experimental material used in this study was *T. amurensis*. Fresh tender leaves were collected from a *T. amurensis* tree (36.627414N, 117.166440E; 210 m above sea level) located at the Shandong Provincial Forestry and Grassland Germplasm Resources Center in September 2023. The collected samples were immediately immersed in liquid nitrogen for rapid freezing and then stored in an ultra-low temperature refrigerator at -80°C.

Genomic DNA was extracted using a DNA extraction kit (TIANGEN, China) and its quality was verified by both ultraviolet spectrophotometry and agarose gel electrophoresis. Sequencing libraries were constructed separately for second-generation and third-generation sequencing. The second-generation sequencing library was prepared by randomly shearing genomic DNA using a Covaris ultrasonicator. The workflow included end repair, A-tailing, adapter ligation, purification, and PCR amplification. Library quantification was performed using Qubit, fragment size distribution was assessed on an Agilent 2100 Bioanalyzer, and accurate quantification for sequencing was achieved by Q-PCR. Finally, the library was sequenced on an Illumina NovaSeq 6000 platform with a depth of 188× coverage (Su X., 2024). The third-generation sequencing library was prepared following the standard Oxford Nanopore Technologies (ONT) protocol using the SQK-LSK109 kit (Bolger et al., 2014). The procedure included DNA damage repair, end repair, and adapter ligation steps, performed without PCR amplification. Sequencing was carried out on an Oxford Nanopore platform, yielding a final depth of 100× coverage. Upon obtaining the raw data, quality control was performed on the short-read and long-read datasets using Fastp and NanoPlot, respectively. Only reads with a quality score (Q) ≥ 19 were retained for subsequent assembly.

### 2.2 Genome assembly and annotation

The mitochondrial genome reads of *T. amurensis* were obtained through sequencing and quality screening. Using long-read data, we performed a preliminary assembly with the Flye software (v2.9, parameters: –nano-hq) (Kolmogorov et al., 2019), which generated graphical assembly results in Graphical Fragment Assembly (GFA) format. Build the database using makeblastdb, and use the Nucleotide Basic Local Alignment Search Tool (BLASTn) program (parameters: -evalue 1e-5 -outfmt 6 -max_hsps 10 -word_size 7 -task blastn-short) to identify the contig fragments containing mitochondrial genomes. The GFA files were visualized using the Bandage software (v0.8.1) (Wick et al., 2015). Contigs corresponding to the mitochondrial genome were screened based on the BLASTn results, yielding a draft genome for *T. amurensis*. The mitochondrial reads were extracted by aligning both long and short reads to the obtained mitochondrial contigs using the MEM algorithm of the Burrows-Wheeler Aligner (BWA, v0.7.17) (Li and Durbin, 2009). The complete mitochondrial genome was then generated through a hybrid assembly approach performed in Unicycler (parameters: –mode normal) (Wick et al., 2017).

Genome annotation was conducted using Geseq software (v2.03, parameters: –circular –evalue 1e-5) (Michael et al., 2017) and IPMGA (http://www.1kmpg.cn/ipmga/). tRNA genes were annotated using tRNAscan-SE (v2.0.11, parameters: -O –organellar) (Lowe and Eddy, 1997), while rRNA genes were identified using BLASTn (v2.13.0, parameters: -evalue 1e-10 -perc_identity 80) (Chen et al., 2015). All annotation errors were manually corrected with Apollo software (v1.11.8) (Lewis et al., 2002).

The complete assembly and annotation data have been uploaded to the National Center for Biotechnology Information GenBank database. The accession number is PQ072837 for the *T. amurensis* mitochondrial genome (https://www.ncbi.nlm.nih.gov/nuccore/PQ072837).

### 2.3 Codon usage preference and RNA editing site analysis

The protein-coding sequences of the mitochondrial genome were extracted using PhyloSuite software (v1.1.16) (Zhang et al., 2020). The codon usage preferences were analyzed using MEGA software (v7.0) (Kumar et al., 2016), and the Relative Synonymous Codon Usage (RSCU) values were calculated. Codons with RSCU >1 were considered preferentially used codons, whereas those with RSCU <1 were regarded as less preferred. An RSCU = 1 indicated no usage bias.

The sequences of all protein-coding genes (PCGs) encoded by the mitochondrial genome were used to predict RNA editing sites. RNA editing from cytosine (C) to uracil (U) in mitochondrial PCGs was predicted using Deepred-mt (Edera et al., 2021), and only sites with probability values greater than 0.9 were retained for further analysis.

### 2.4 Analysis of repetitive sequences and homologous sequence transfer

Repetitive sequences are identical or complementary fragments that occur at different positions in the genome. These repetitive sequences play an important role in gene regulation. The Tandem Repeats Finder software (v4.09) (https://tandem.bu.edu/trf/trf.unix.help.html) (Benson, 1999) and REPuter web server (https://bibiserv.cebitec.uni-bielefeld.de/reputer/) (Stefan et al., 2001) were used to identify various repeat types, including palindromic, complementary, forward, and reverse repeats in the mitochondrial genome of *T. amurensis*. MISA (v2.1) (https://webblast.ipk-gatersleben.de/misa/) (Beier et al., 2017) was used to identify SSRs, with manual correction of the identified SSR loci. The results were visualized using Microsoft Excel (2021) and the Circos package (v0.69.9) (Zhang et al., 2013).

Homologous fragments were analyzed using the BLASTn (v2.13.0) (Chen et al., 2015), and the results were visualized using the Circos package (v0.69.9) (Zhang et al., 2013).

### 2.5 Collinearity and phylogenetic analysis

Pairwise BLASTn comparisons of mitochondrial genomes were conducted. Homologous sequences exceeding 500 bp were retained and defined as conserved collinear blocks for the construction of the Multiple Synteny Plot.

Mitochondrial genome sequences of 35 species, including *T. amurensis*, were obtained from the GenBank database (**Supplementary Table S1**). These comprised 15 species from the Malvaceae family (8 *Gossypium*, 1 *Hibiscus*, 1 *Abelmoschus*, 1 *Bombax*, 2 *Corchorus*, and 2 *Theobroma*), 14 species from the Brassicaceae family (4 *Brassica*, 1 *Eruca*, 1 *Raphanus*, 1 *Rhamphospermum*, 1 *Schrenkiella*, 1 *Arabis*, 2 *Capsella*, 2 *Arabidopsis*, and 1 *Cochlearia*), and one species each from the following families: Thymelaeaceae (*Aquilaria*), Bataceae (*Batis*), Caricaceae (*Carica*), Rutaceae (*Citrus*), and Meliaceae (*Toona*).

Phylogenetic analysis was performed employing a set of 25 conserved protein-coding genes (*atp*6, *atp*8, *atp*9, *ccm*B, *ccm*C, *ccm*FC, *cob*, *cox*1, *cox*3, *mat*R, *mtt*B, *nad*1, *nad*2, *nad*3, *nad*4, *nad*5, *nad*6, *nad*7, *nad*9, *rpl*2, *rpl*5, *rpl*16, *rps*3, *rps*14, and *rps*19), resulting in the construction of a phylogenetic tree. Common genes were extracted using PhyloSuite (v1.1.16) (Zhang et al., 2020), and multiple sequence alignments of the mitochondrial genomes were performed using MAFFT software (v7.505) (Katoh and Standley, 2013). A phylogenetic tree was constructed using the maximum likelihood method in IQ-TREE software (v1.6.12) (Nguyen et al., 2015), with 1000 ultrafast bootstrap replicates (–alrt 1000) and 1000 standard bootstrap replicates (-b 1000). Finally, the results of the phylogenetic analysis were visualized using the Interactive Tree of Life software (v6) (Letunic and Bork, 2019).

## 3 Results

### 3.1 Structural characteristics of the mitochondrial genome of *T. amurensis*

**Figure 1:**
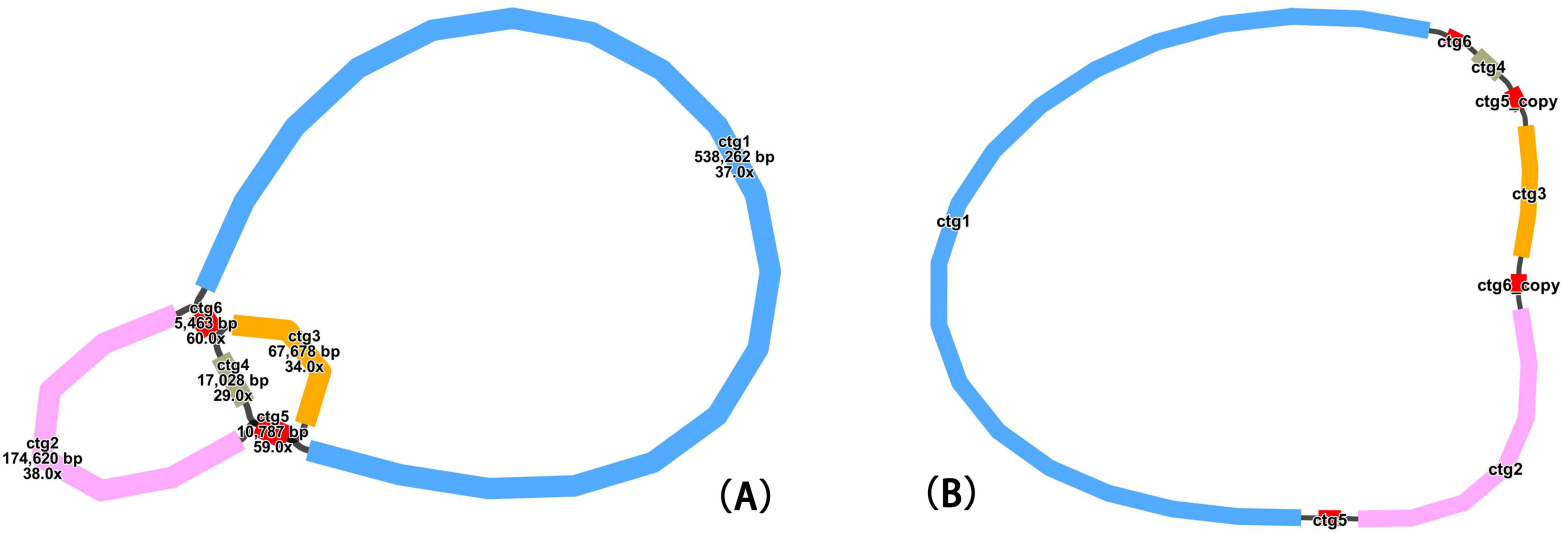
**(A)** Sketch of the mitochondrial genome of *Tilia amurensis*. **(B)** Circular conformation of the master mitochondrial genome.

Through software visualization, a sketch of the mitochondrial genome of *T. amurensis* was assembled based on long-read sequencing data (**Figure 1A**). The assembly consisted of six nodes in total (**Supplementary Table S2**). After excluding duplicate regions, one primary circular contig was obtained (**Figure 1B**). The main structure of the *T. amurensis* mitochondrial genome consisted of a single circular molecule, with a total length of 830,088 bp and a GC content of 44.97%. Analysis revealed the presence of two pairs of repetitive sequences that potentially mediate alternative recombination conformations, indicating the existence of multiple structural configurations of the mitochondrial genome (**Supplementary Figure S1**).

**Figure 2:**
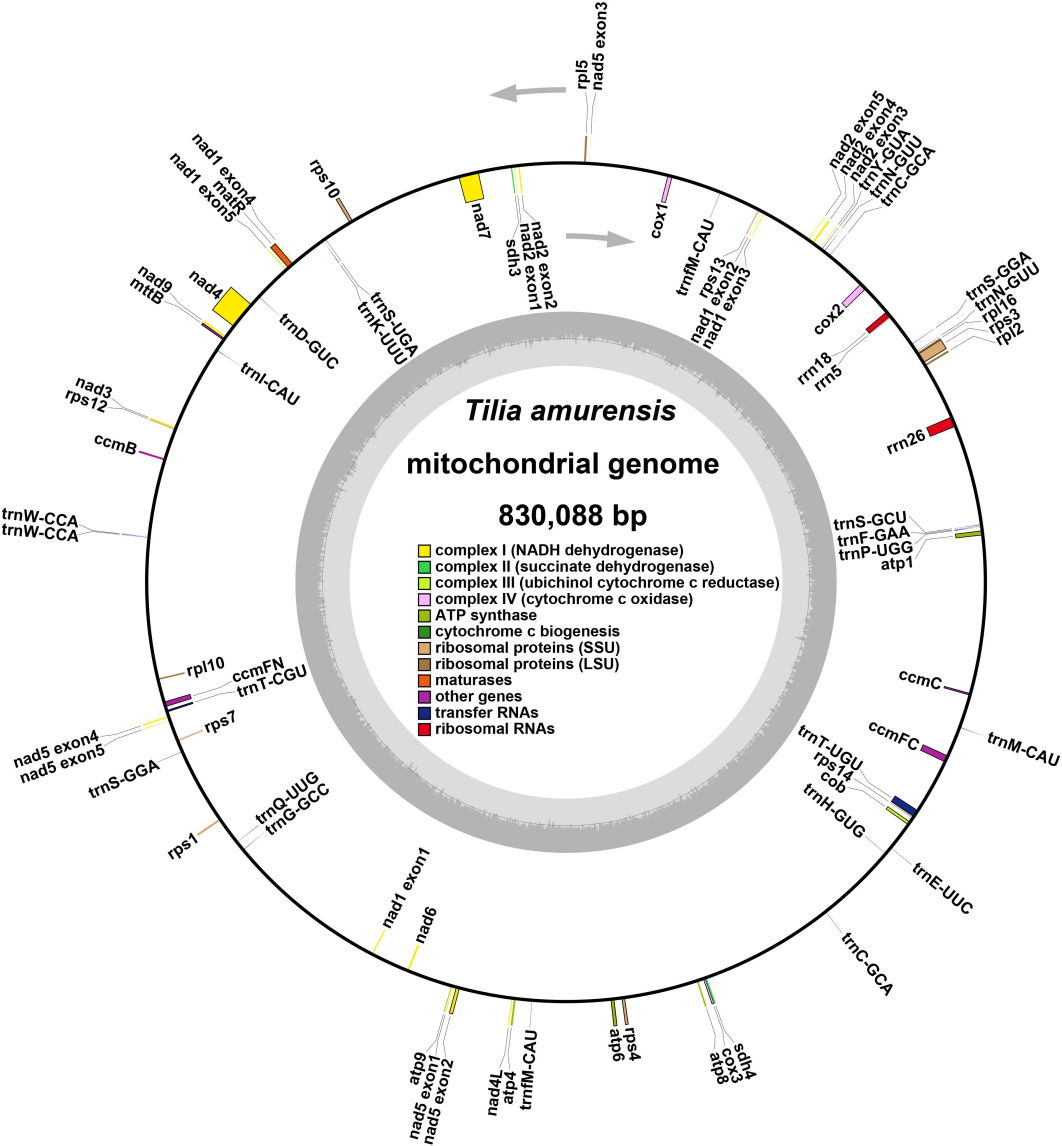
Diagram representing the annotated circular structure of *T. amurensis* mitochondrial genome.

A total of 61 genes were annotated in the circular mitochondrial genome of *T. amurensis*, including 20 tRNA genes, 3 rRNA genes, and 38 PCGs (**Figure 2**, **Supplementary Table S3**). Based on their function, the genes were classified into nine categories: ATP synthase (five genes: *atp*1*, atp*4*, atp*6*, atp*8, and *atp*9); NADH dehydrogenase (nine genes: *nad*1*, nad*2*, nad*3*, nad*4*, nad*4L*, nad*5*, nad*6*, nad*7, and *nad*9); Cytochrome b (one gene: *cob*); Cytochrome c biogenesis (four genes: *ccm*B*, ccm*C*, ccm*FC, and *ccm*FN); Cytochrome c oxidase (three genes: *cox*1*, cox*2, and *cox*3); maturase (one gene: *mat*R); protein transport subunit (one gene: *mtt*B); ribosomal proteins (twelve genes: *rpl*2*, rpl*5*, rpl*10, and *rpl*16*; rps*1*, rps*3*, rps*4*, rps*7*, rps*10*, rps*12*, rps*13, and *rps*14); succinate dehydrogenase (two genes: *sdh*3 and *sdh*4).

### 3.2 Codon usage and RNA editing site analysis

Amino acids are encoded by either a single codon (e.g., tryptophan [Trp], UGG) or multiple codons (e.g., alanine [Ala], GCU, GCA, GCC, GCG). The usage frequency of these codons in mitochondrial genomes varies significantly across species, reflecting codon usage bias.

**Figure 3:**
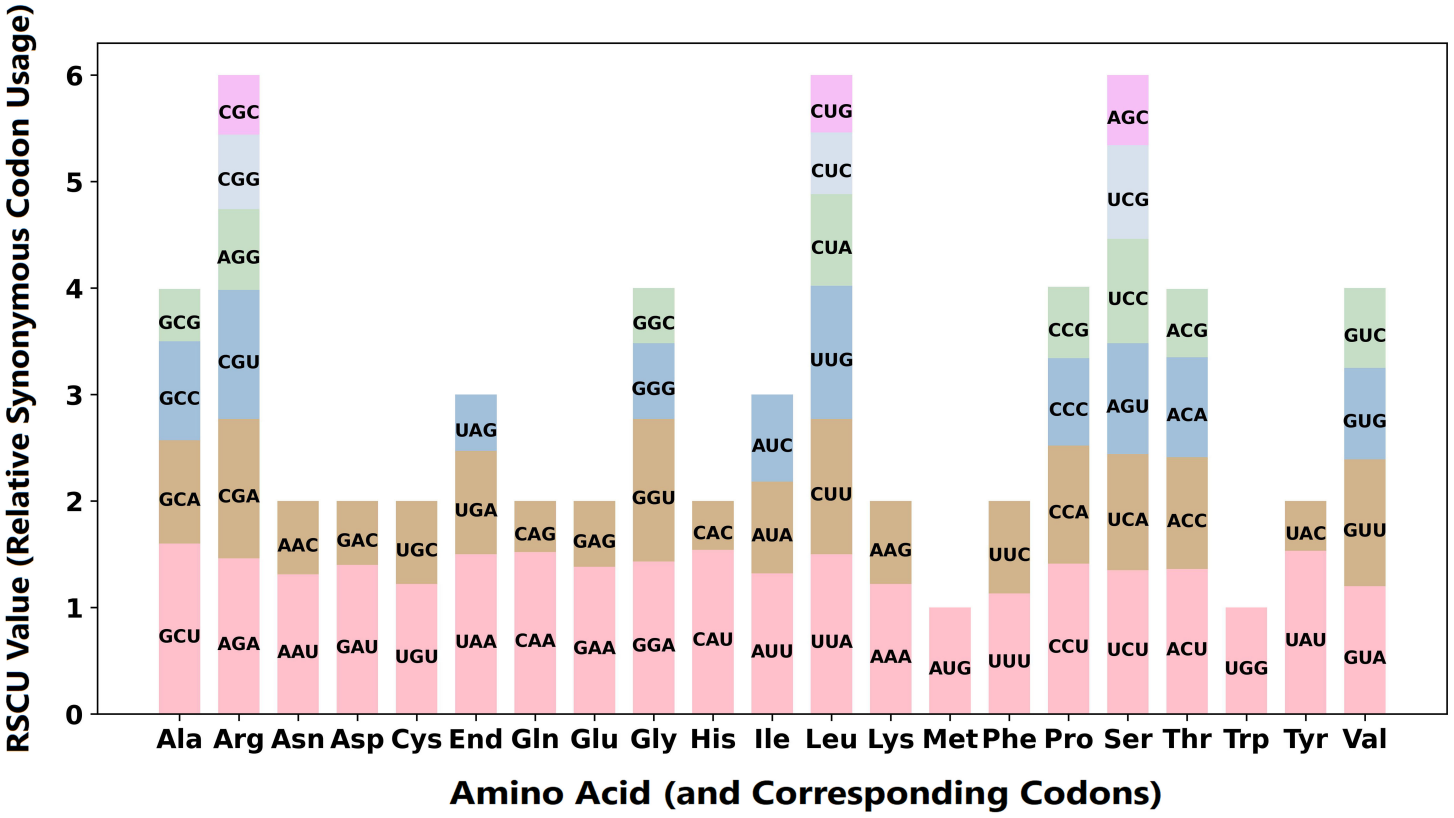
Codon usage preference analysis in the *T. amurensis* mitochondrial genome.

Codon preference analysis was conducted on the 38 PCGs in *T. amurensis* mitochondrial genome (**Figure 3**, **Supplementary Table S4**). Nine amino acids were encoded by only two codons: asparagine (Asn), aspartic acid (Asp), cysteine (Cys), glutamine (Gln), glutamic acid (Glu), histidine (His), lysine (Lys), phenylalanine (Phe), and tyrosine (Tyr). These amino acids display codon preference, that is, the RSCU value is >1. The start codons AUG and UGG both exhibit an RSCU value of 1.00. Among all amino acids, Ala has the strongest preference for the GCU codon (RSCU = 1.60), while His shows the strongest preference for the CAU codon (RSCU = 1.54). Conversely, His shows the weakest preference for the CAC codon (RSCU = 0.46), while Tyr has the weakest preference for the UAC codon (RSCU = 0.47).

RNA editing is a post-transcriptional modification process. It is widely present in all eukaryotes. However, the number and types of RNA editing sites vary across species, including *T. amurensis*.

**Figure 4:**
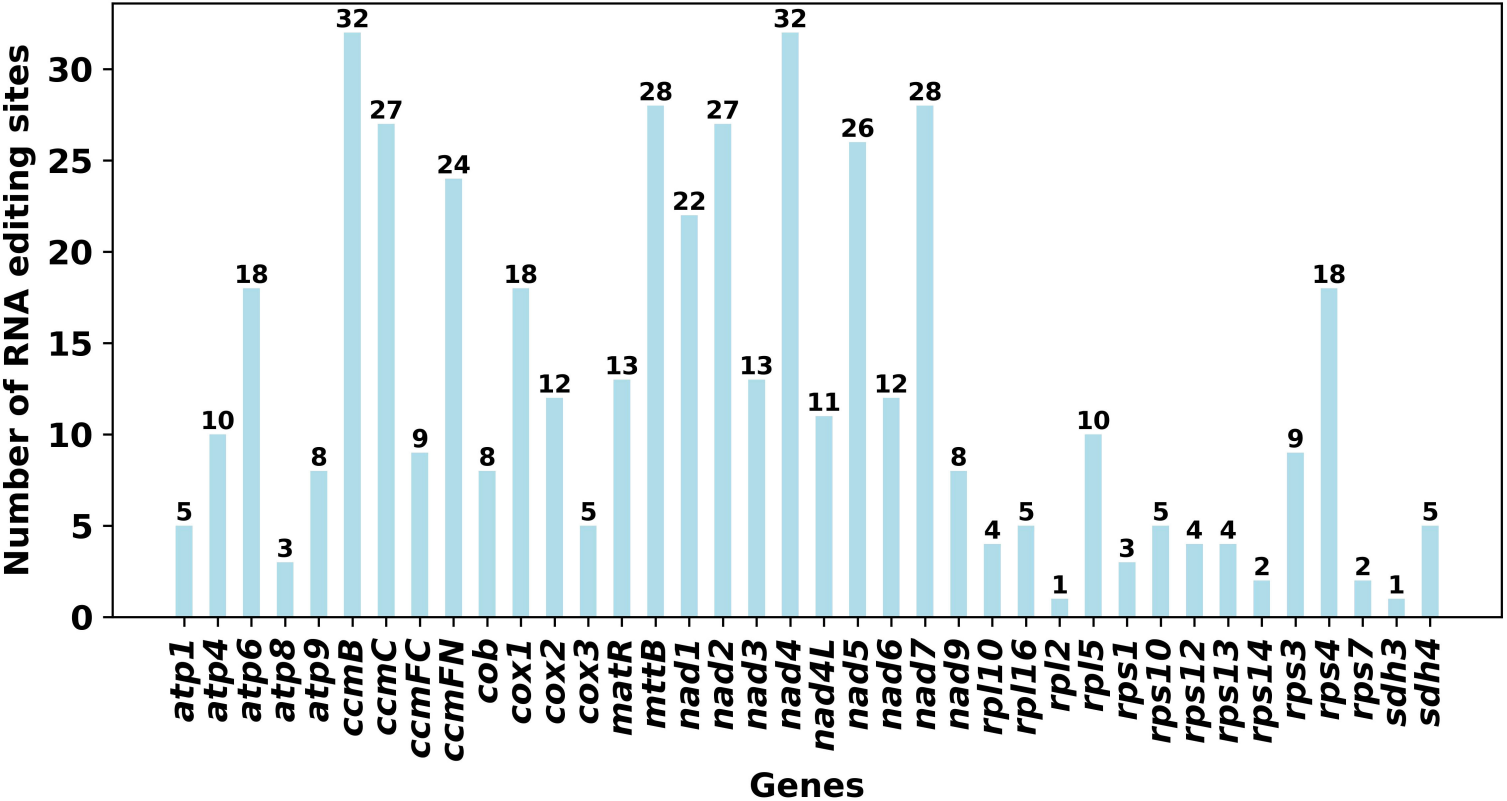
Distribution and number of RNA editing sites in *T. amurensis* mitochondrial genome.

As shown in **Figure 4**, a total of 472 RNA editing sites were identified within the 38 PCGs. Among these coding genes, *ccm*B and *nad*4 contained the largest number of potential editing sites (32 sites each), followed by *mtt*B and *nad*7 (28 sites each). In contrast, *rpl*2 and *sdh*3 had the fewest (one site each). Similar to most angiosperms, an abundance of RNA editing sites is conducive to maintaining the stability of the mitochondrial respiratory chain in plants (Su, 2024).

### 3.3 Analysis of repetitive and homologous transferred sequences

**Figure 5:**
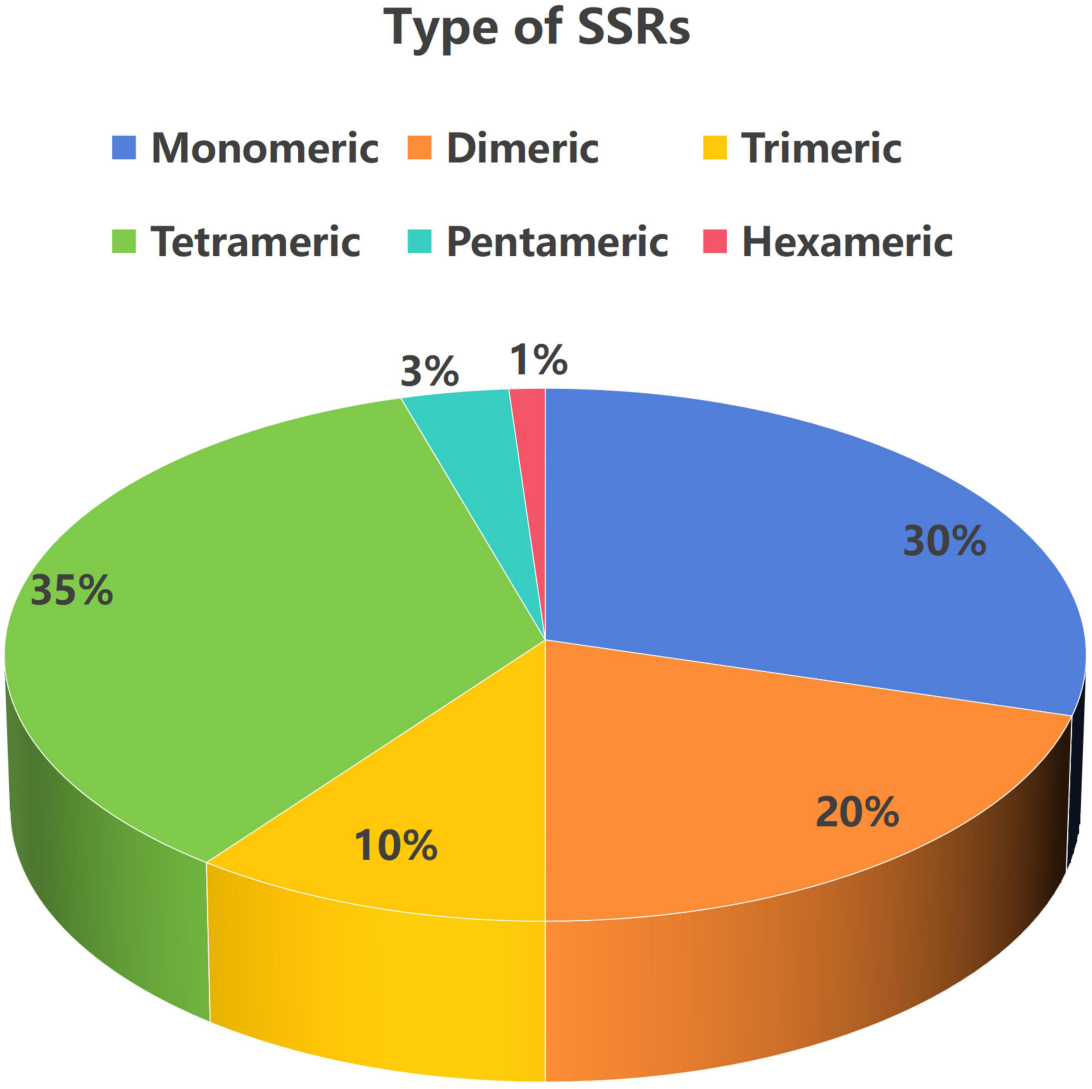
Proportion of various types of simple sequence repeats in *T. amurensis* mitochondrial genome.

SSRs are short DNA fragments in the genome composed of repeating units of 1–6 bases, serving as common and effective molecular markers (Atul and Sharma, 2014). A total of 264 eligible SSRS were identified in the mitochondrial genome of *T. amurensis*, including 78 monomers, 54 dimers, 27 trimers, 93 tetramers, 9 pentamers, and 3 hexamers. As shown in **Figure 5**, tetrameric repeats were the most abundant, accounting for 35.00% of all SSR types, while monomeric and dimeric repeats accounted for 50.00%. Thymine (T) monomer repeats accounted for 57.69% (45 occurrences) of all monomer repeats.

**Figure 6:**
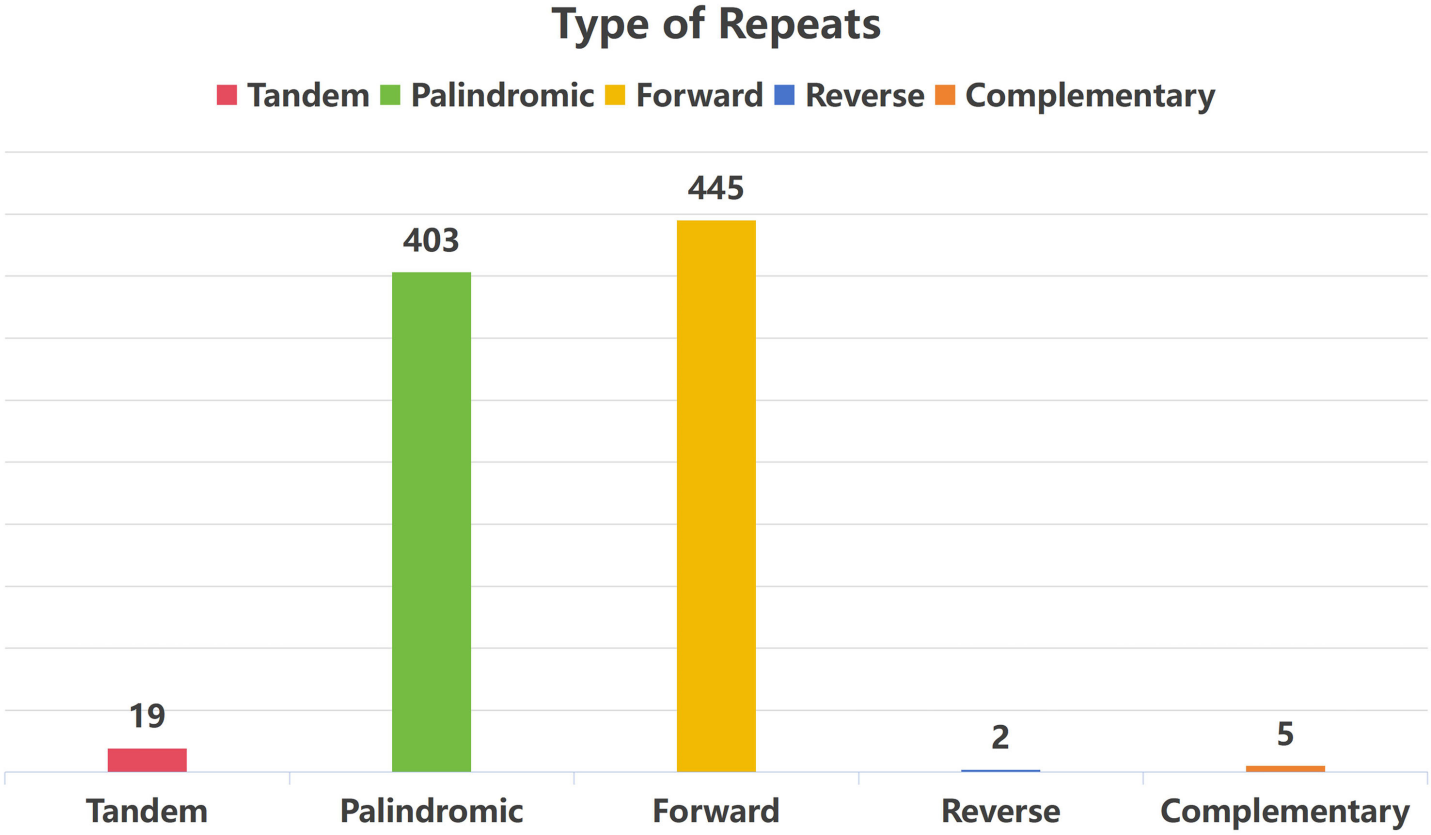
Bar chart of the repetitive sequence distribution in *T. amurensis* mitochondrial genome.

Tandem repeat sequences (also known as satellite DNA) refer to core repeat units of 7–200 bases that are tandem and repeated multiple times. They are generally classified as long, short, and medium types (Mishra and Sahoo, 2021). Nineteen long tandem repeat sequences were located in the mitochondrial genome of *T. amurensis*, with a sequence identity >81% and lengths ranging 15–28 bp. Additionally, 855 pairs of scattered repetitive sequences with lengths ≥30 bp were located. These included 403 pairs of palindromic repeats (with the longest spanning 5,463 bp), 445 pairs of forward repeats (the longest reaching 10,787 bp), 2 pairs of reverse repeats, and 5 pairs of complementary repeats (**Figure 6**).

**Figure 7:**
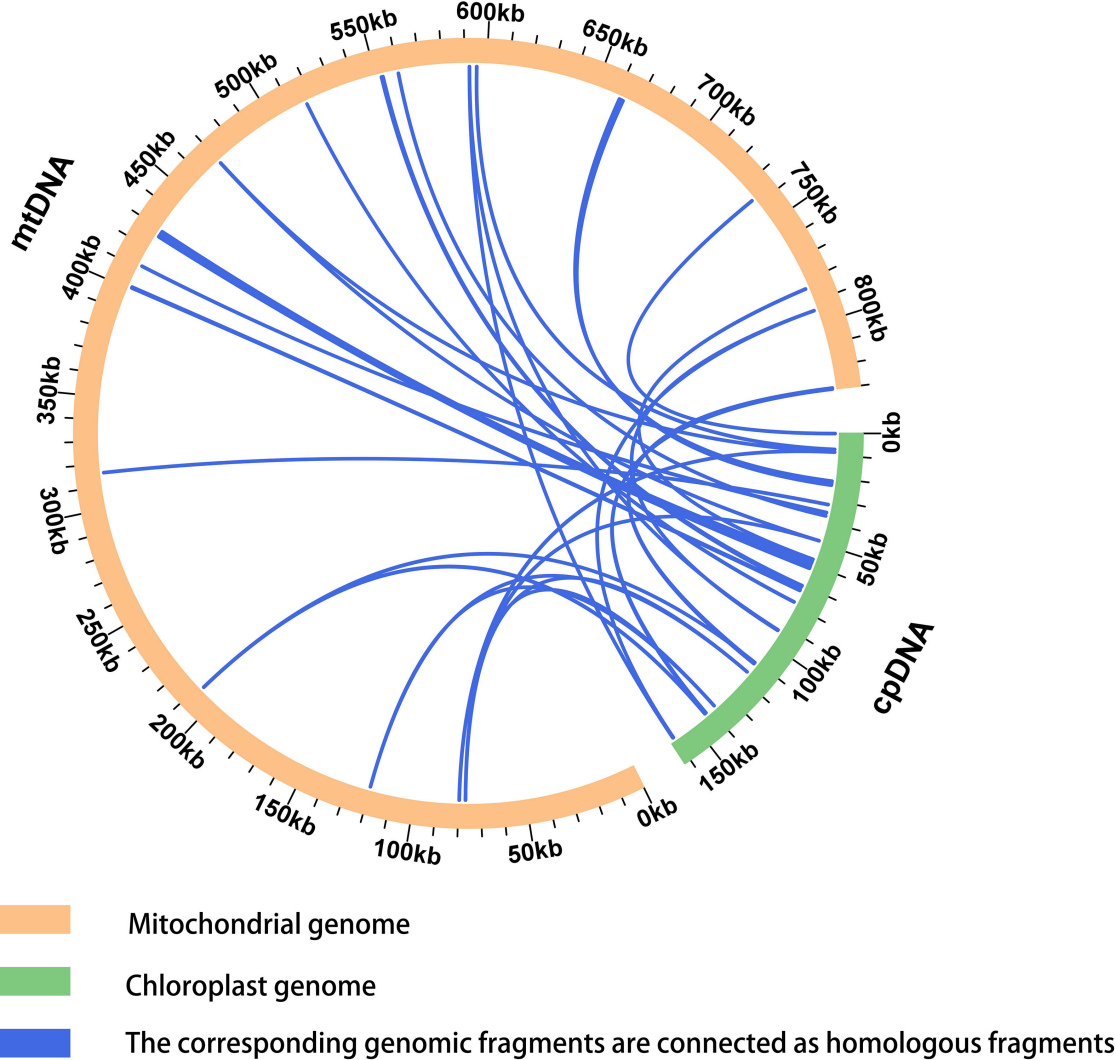
Analysis of homologous sequence transfer in *T. amurensis* mitochondrial genome.

Sequence similarity analysis (**Figure 7**, **Supplementary Table S5**) identified 22 homologous chloroplast-derived fragments with a total length of 8,138 bp, accounting for 0.98% of the total mitochondrial genome length. Among these fragments, the longest, mitochondrial plastid DNA transfer (MTPT) 4, reached 1,887 bp. Gene annotation of these homologous regions revealed 10 complete genes distributed within these fragments, including two PCGs (*psb*F and *psb*L) and eight tRNA genes (*trn*D-GUC, *trn*H-GUG, *trn*I-CAU, *trn*M-CAU, *trn*N-GUU, *trn*P-UGG, *trn*S-GGA, and *trn*W-CCA).

### 3.4 Synteny and phylogenetic analysis

**Figure 8:**
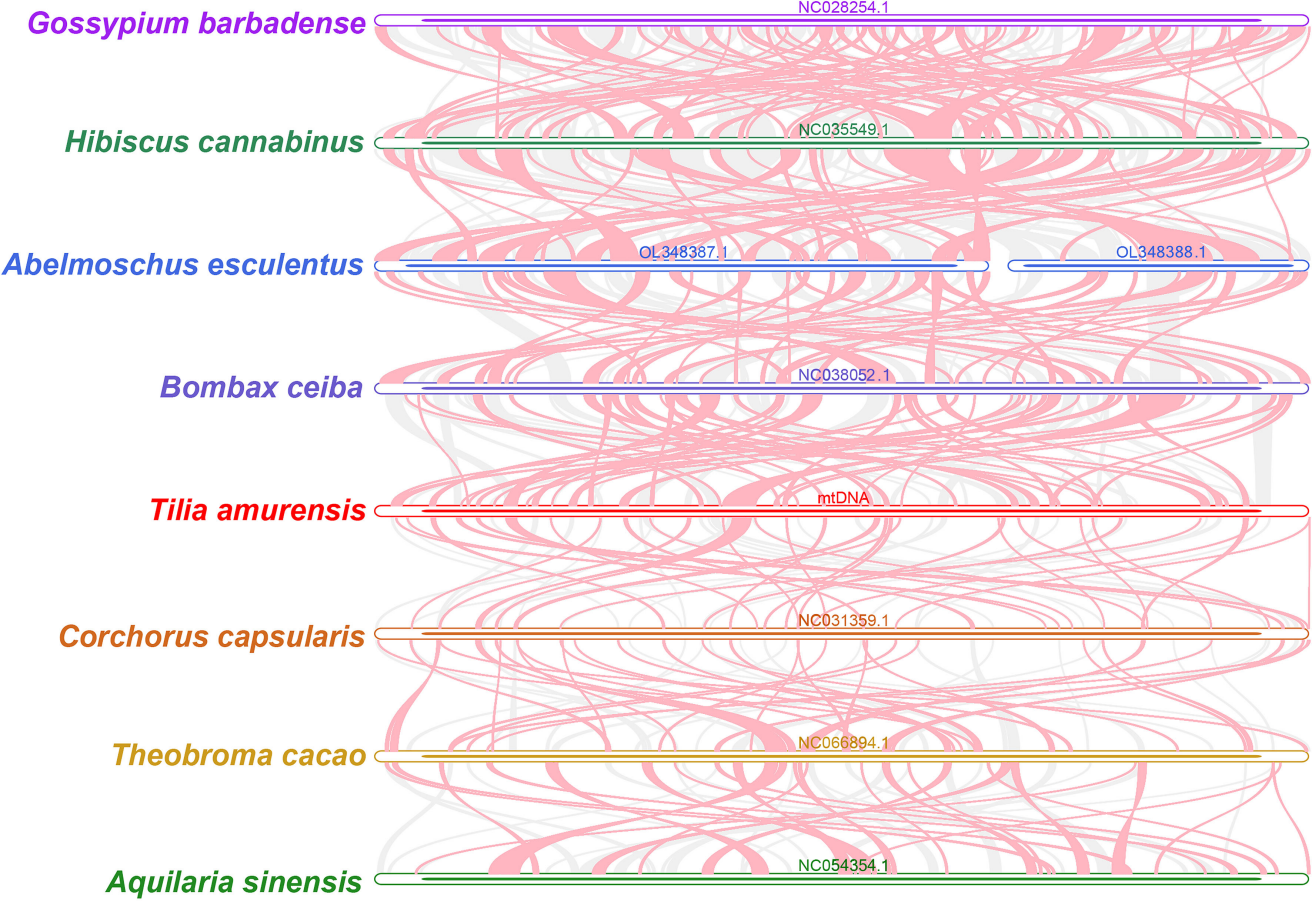
Homology analysis of mitochondrial genomes. Red arcs indicate regions of inversion; gray areas indicate regions with good homology. Collinear blocks with lengths <0.5 kb were excluded from the results.

To investigate the sequence structure and evolutionary dynamics of mitochondrial genomes within the order Malvales, a comparative analysis was conducted on the mitogenomes of eight closely related species (**Figure 8**). The results revealed that the mitochondrial genomes of these species are connected by numerous homologous sequence blocks, yet overall synteny is remarkably low. Notably, although the *T. amurensis* genome contains abundant short homologous regions—indicating shared ancestral sequences with its relatives—it also harbors a considerable number of unique non-conserved regions that lack sequence homology in the other species examined.

More strikingly, the synteny plot clearly demonstrates a high degree of rearrangement in the order of conserved homologous blocks among these species, reflecting substantial reorganization of genomic architecture. These findings provide strong evidence that *T. amurensis* has undergone significant genomic rearrangement during its evolutionary history. Moreover, as this pattern is observed across multiple species in Malvales, it reveals a highly dynamic structure of mitochondrial genomes in this plant order, characterized by frequent recombination events that have led to the extensive non-conservation of sequence arrangement observed today.

**Figure 9:**
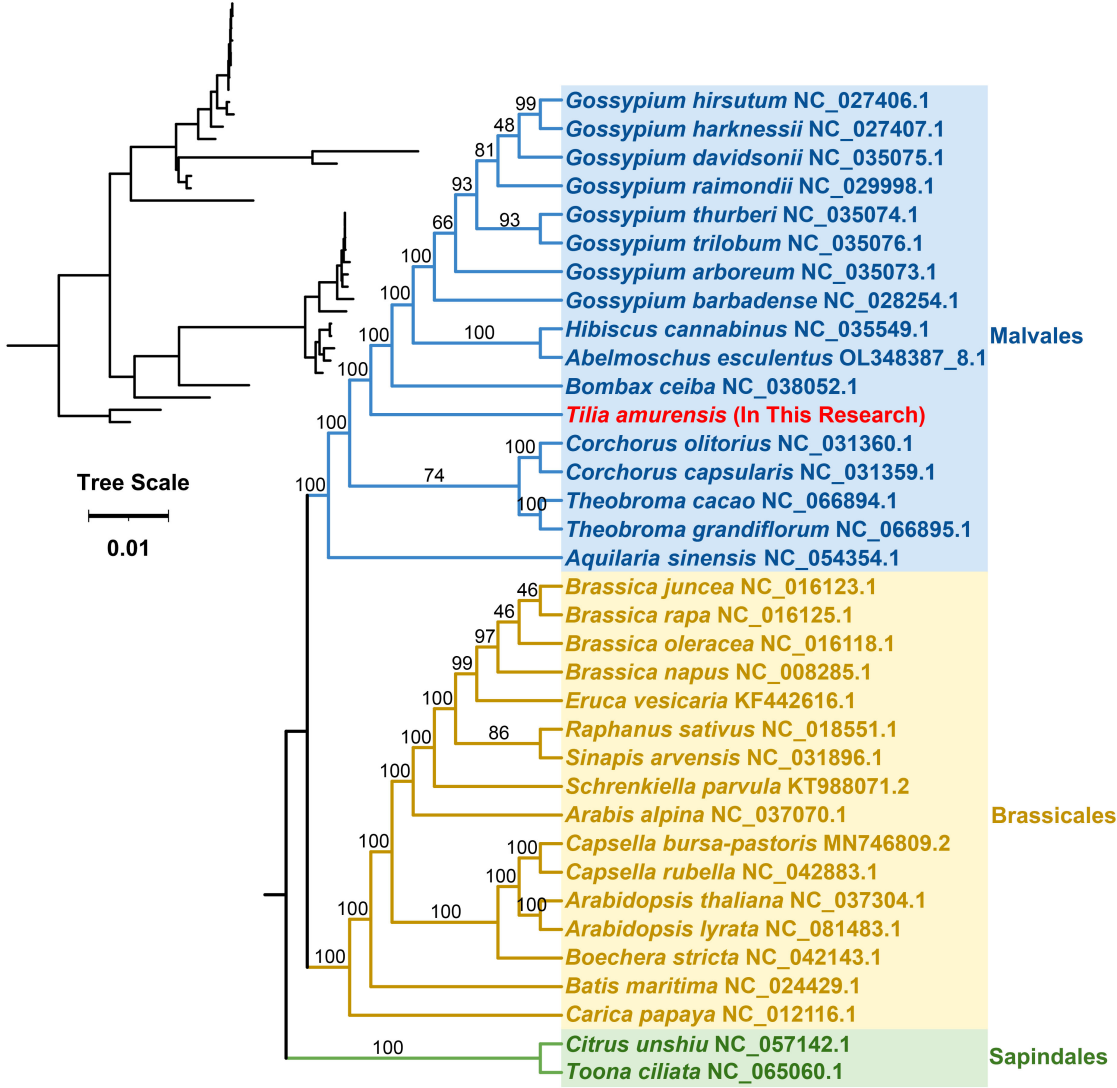
Phylogenetic analysis of *T. amurensis* and related species based on conserved mitochondrial genes.

This study downloaded the mitochondrial genomes of 35 species from three orders—Malvales, Sapindales, and Brassicales—including *T. amurensis*, from the GenBank database. A comparative analysis of insertions, deletions, and repeats in these sequences revealed the complex evolutionary dynamics of plant mitochondrial genomes. To further explore evolutionary patterns, we referred to mitochondrial genome sequences of these plant species. A phylogenetic tree was constructed using DNA sequences of 25 conserved protein-coding genes (PCGs), with two species from Sapindales designated as the outgroup (**Figure 9**).

The resulting topology is highly consistent with the latest Angiosperm Phylogeny Group (APG) classification: species from Malvales, Sapindales, and Brassicales each formed strongly supported monophyletic clades (key node bootstrap value = 100), confirming the placement of *T. amurensis* within the Malvaceae family under Malvales. Within these orders, the tree also clearly resolved the evolutionary relationships among species, particularly the affiliations of *T. amurensis* with its close relatives in Malvales. However, although the tree suggests a closer relationship between Malvales and Sapindales, the deep node uniting these three orders received low bootstrap support (10), indicating that their exact phylogenetic relationships require further investigation with additional data.

## 4 Discussion

In the cells of higher plants, the chloroplast and mitochondrial genomes are considered cytoplasmic genomes, often with a semi-autonomous mode of inheritance. Compared with the chloroplast genome, the plant mitochondrial genome is larger, experiences notable recombination (Su, 2024), and exhibits substantial variation in features such as total base length, gene content, gene arrangement sequence, CG content, and overall genomic structure (Richardson et al., 2013; He et al., 2023). Most mitochondrial genomes are composed of a single circular molecule, although a few plant species exhibit linear or multi-circular structures (Su et al., 2024). *T. amurensis* is a common deciduous tree of the Malvaceae family. However, mitochondrial genome data within Malvaceae remain scarce, and reported genome sizes vary significantly. Xiaofang Liao et al. used Single-Molecule Real-time Sequencing Technology to sequence the complete mitochondrial genome of *Hibiscus cannabinus* L. in 2018, reporting a total genome length of 569,915 bp (Liao et al., 2018). In the same year, Yong Gao et al. studied *Bombax ceiba* L using the PacBio sequencing platform, obtaining a total mitochondrial genome length of 594,390 bp (Gao et al., 2018). In 2022, Jihan Li reported the mitochondrial genome of *Abelmoschus esculentus* (L.) Moench, which consists of two independent mitochondrial DNA molecules. One of the molecules has a complex, multi-branched but closed circular structure, with a total length of 355,223 bp. The other molecule presents a typical circular conformation (174,444 bp) (Li et al., 2022). In contrast, the mitochondrial genome of *T. amurensis* assembled in this study was larger, with a total length of 830,088 bp. Its size exceeds that of the three aforementioned species, and its structural organization is similar to that of *H. cannabinus* and *B. ceiba*, characterized by a single circular molecule.

Codon usage bias is a significant feature in eukaryotic genome evolution, with substantial variation among species measured by the RSCU index (Deng et al., 2012). In the mitochondrial genome of *T. amurensis*, the RSCU values of the start codon AUG (methionine [Met]) and UGG (Trp) were both 1. In contrast, the stop codons UAA and UAG had significantly different usage frequencies, with RSCU values of 1.50 and 0.53, respectively. In the mitochondrial genome of *H. cannabinus*, the RSCU value of the start codon AUG is 2.98. Its usage frequency is significantly higher than that of other synonymous codons (such as CUG and UUG), and also higher than that of *T. amurensis*. This indicates that the *H. cannabinus* mitochondrial gene may be subjected to strong selection pressure at the initiation stage of translation (Liao et al., 2018). In *A. esculentus*, the usage frequency of the stop codon UGA (1.42) was higher than that of UAA (0.88), differing from most higher plants. This pattern may reflect a species-specific adaptation in translation termination (Li et al., 2022). Among all amino acids in the mitochondrial genome of *T. amurensis*, Ala showed the strongest preference for the GCU codon, while His showed the strongest preference for the CAU codon, with RSCU values of 1.60 and 1.54, respectively. Conversely, His had the weakest preference for the CAC codon, while Tyr had the weakest preference for the UAC codon, with RSCU values of 0.46 and 0.47, respectively. In the *H. cannabinus* mitochondrial genome, the stop codons UAA and GCU (Ala) showed relatively high usage frequencies, with RSCU values of both being 1.63. In *B. ceiba*, Leu is more frequently encoded by UUA and CUU (RSCU = 1.89 and 1.45, respectively), while CUC and CUG are used less frequently (RSCU = 0.32 and 0.18). This pattern may be related to the abundance and translation efficiency of mitochondrial tRNA (Gao et al., 2018). For Trp, *B. ceiba* is consistent with *T. amurensis* and is encoded only by UGG, with an RSCU value of 1. However, in *A. esculentus*, Phe showed a significant preference for UUU (1.52) over UUC (0.48), while Asn preferred AAU (1.68) over AAC (0.32). These results indicate that UUU and AAU might be the dominant codons in the *A. esculentus* mitochondrial genome. Overall, the codon usage patterns of PCGs in four Malvaceae species demonstrated clear preferences, such as high usage of the stop codon UAA, which is consistent with the mitochondrial genomes of many higher plants. However, the UGA preference of *A. esculentus* may represent a unique evolutionary adaptation (Li et al., 2022). Additionally, in the mitochondrial genome of most Malvaceae plants, the AUG codon for Met typically has a high RSCU value (>2.5), except in *T. amurensis*. In contrast, the UGG codon for Trp (RSCU = 1) remains highly conserved, indicating strong functional constraints on these codons.

Plant mitochondrial genomes harbor diverse repetitive sequences—such as tandem, simple, inverted, and dispersed repeats—which facilitate homologous recombination (Guo et al., 2017). This recombination frequently produces multiple genomic conformations and plays a key role in driving structural variation and evolution of the mitochondrial genome (Ye et al., 2017; Morley and Nielsen, 2017). A comparative analysis of repetitive sequence characteristics among three Malvaceae species revealed notable differences. In *T. amurensis*, 264 SSRS, 19 tandem repetitions, and 855 pairs of scattered repetitions were identified, predominantly comprising short fragment repetitions. In contrast, *H. cannabinus* harbored 584 repeat sequences (95% of which ranged 20–100 bp) and possessed three large fragment repeats exceeding 1 kb (with the longest spanning 7,782 bp). These large fragment repeats may drive genomic rearrangement via homologous recombination, accounting for 11.71% of the total genome length (Liao et al., 2018). Among the 14 pairs of recombination-related repeat sequences identified in *A. esculentus*, three pairs of long repeats (such as the 15,128 bp LR9) mediate high-frequency recombination, with an isomeric ratio of 48:52. The remaining 11 pairs of short repeats generally exhibit recombination frequencies below 2%. However, four pairs (SR2, SR4, SR7, and SR8) are capable of mediating low-frequency inter-molecular recombination, offering new insights into the multi-molecular structural mechanisms of plant mitochondria (Li et al., 2022). Previous studies have shown that the length of repetitive sequences is positively correlated with the recombination frequency, and large fragment repetitions are more likely to cause genomic structural variations. This trend is consistent across the three species examined; however, the scale, distribution, and function of repetitive sequences differ significantly across species. This reflects the diversity and complexity of mitochondrial genome evolution in plants.

During mitochondrial evolution, some chloroplast fragments have migrated into the mitochondrial genome, and the length and sequence similarity of these migrated fragments vary among different species (Cheng et al., 2021; Zhai et al., 2024). Sequence transfer from chloroplast genomes was found in the mitochondrial genomes of all four Malvaceae species. A comparative analysis of the mitochondrial plastid DNA transfers (MTPTs) in the mitochondrial genomes of the four Malvaceae species revealed that *T. amurensis* contained 22 fragments (8138 bp, accounting for 0.98%), with the longest being 1887 bp. These fragments included 10 complete genes, such as *psb*F and *psb*L. *H. cannabinus* contained 15 fragments (11,281 bp, 1.98%), including complete genes such as *psa*A and *ndh*B, and 8 fragments related to tRNA genes, indicating a tendency for cross-organelle transfer of tRNA genes (Liao et al., 2018). The migration phenomenon was most pronounced in *A. esculentus*, which harbored 28 fragments (21,231 bp), among which 6 exceeded 1 kb (the longest being 5,142 bp). The fragments included genes such as *psa*A and *rps*7 and the *psb*J-F-E-L pseudogene cluster. However, no expression was detected in the transcriptome, indicating functional loss (Li et al., 2022). Altogether, these findings indicate significant interspecific differences in the length and quantity of MTPTs. tRNA and photosynthesis-related genes (such as members of the *psb* and *psa* families) are more prone to migration. Large-scale fragment transfers often involve gene clusters, but are frequently accompanied by pseudogenization. Functional retention varies across species. The gene silencing phenomenon in *A. esculentus* reveals the selective pressure of mitochondria on migratory sequences. These findings provide important insights into the evolutionary dynamics of gene transfer between organelles.

Collinearity analysis examines the arrangement of homologous genes or sequences, revealing the structural features and evolutionary dynamics of the mitochondrial genome in Malvaceae plants. The findings indicate that *T. amurensis* exhibits significant rearrangement characteristics compared with closely related species. Its collinear blocks are short, disordered, and interspersed with a large number of unique sequences, indicating that this species has undergone frequent genomic recombination. *H. cannabinus* has four highly conserved gene clusters, which are widely retained among plant mitochondria and reflect the differences in conservation among different taxonomic orders (Liao et al., 2018). Although *A. esculentus* shows gene sequence variations, some collinear gene clusters maintain the same transcriptional direction. This suggests the formation of multi-cis-inverted transcriptional units and highlights the combined effect of recombination events and functional constraints (Li et al., 2022). Comparative analysis showed that the core gene clusters (such as *rrn*5*–rrn*18) are highly conserved in the Malvaceae family, while the conserved nature of non-essential gene clusters (such as *rpl*5*–rps*14) is family-specific. Recombination mediated by repeat sequences is the main driving force for gene sequence variations, which is particularly prominent in *T. amurensis*. These findings demonstrate a dynamic evolutionary balance between conserved functional modules and structural rearrangements within the mitochondrial genome.

Phylogenetic analysis based on mitochondrial PCGs provides molecular evidence for the classification of plants in the Malvaceae family. Many studies have constructed phylogenetic trees using conserved PCGs (20–25), including core gene families such as *atp*, *cox*, and *nad*. These analyses consistently produce topologies aligned with the APG classification system, supporting the monophyletic nature of the Malvaceae family. *T. amurensis* is clearly positioned within Malvaceae of the order Malvales. *B. ceiba* and *Gossypium* cluster together (Gao et al., 2018), while *H. cannabinus* shows a 100% support rate for close affinity with *Gossypium* (Liao et al., 2018). Although the tree topologies constructed using different functional genomes (such as complexes I and III) are slightly different, the classification at the family level remains stable. Importantly, phylogenetic development of the chloroplast migration sequence (mtpt14) in *A. esculentus* shows that the mitochondrial and chloroplast origin sequences are significantly differentiated, indicating that the sequence has undergone independent evolution after migration (Li et al., 2022). These results not only validate the traditional classification but also indicate that conserved PCGs are reliable in higher-order classification. Furthermore, gene transfer events can be fine-tuned in topological structures through phylogenetic tracking and analyzing the selective pressure on functional genes, providing multi-dimensional molecular evidence for the study of core evolutionary relationships.

The complete mitochondrial genome of *T. amurensis* generated in this study provides an essential reference for evolutionary studies of the genus *Tilia* and the broader Malvaceae family. Future work should aim to systematically sequence mitochondrial genomes of additional congeneric species. With a comprehensive dataset, a robust phylogenomic framework could be established to clarify the evolutionary relationships within the genus. Furthermore, employing comparative genomics approaches—such as analyzing sequence collinearity, mitochondrial-to-plastid DNA transfer events, and the conservation of RNA editing sites—will help elucidate the key mechanisms driving mitochondrial genome evolution in *Tilia*.

## 5 Conclusions

In this study, the mitochondrial genome of *T. amurensis*, a nationally endangered protected species in China, was sequenced. Following assembly and annotation, the mitochondrial genome of *T. amurensis* was found to be a single circular molecule with a total length of 830,088 bp and a GC content of 44.97%. A total of 61 genes were annotated, including 20 tRNA genes, 3 rRNA genes, and 38 PCGs. Moreover, 472 RNA editing sites were detected among the 38 PCGs. *T. amurensis* contained 264 SSRS, 19 tandem repetitions, and 855 pairs of scattered repetitions, primarily short fragment repetitions. Furthermore, 22 chloroplast homologous fragments (with a total length of 8138 bp) were identified, accounting for 0.98% of the total mitochondrial genome length. The phylogenetic tree constructed based on the mitochondrial genomes of 35 plant species clarifies the evolutionary relationships within the Malvaceae family. Additionally, it provides a more accurate understanding of the taxonomic status and divergence time of *Tilia* within Malvaceae, and offers a molecular basis for the conservation and genetic breeding of the endangered *T. amurensis*.

## Data Availability

The data presented in the study are deposited in GenBank on the NCBI website (https://www.ncbi.nlm.nih.gov/), accession number PQ072837.
