# Supplementary material for: Assembly and analysis of the complete mitochondrial genome of endangered plant Tilia amurensis Rupr
Source: Front Plant Sci. 2025 Dec 3;16:1686403. doi: 10.3389/fpls.2025.1686403 (PMC12708923; doi:10.3389/fpls.2025.1686403)
Supplement: Supplementary file 1 [file Table1.docx]

Supplementary Material

# Supplementary Figures and Tables

## Supplementary Figures


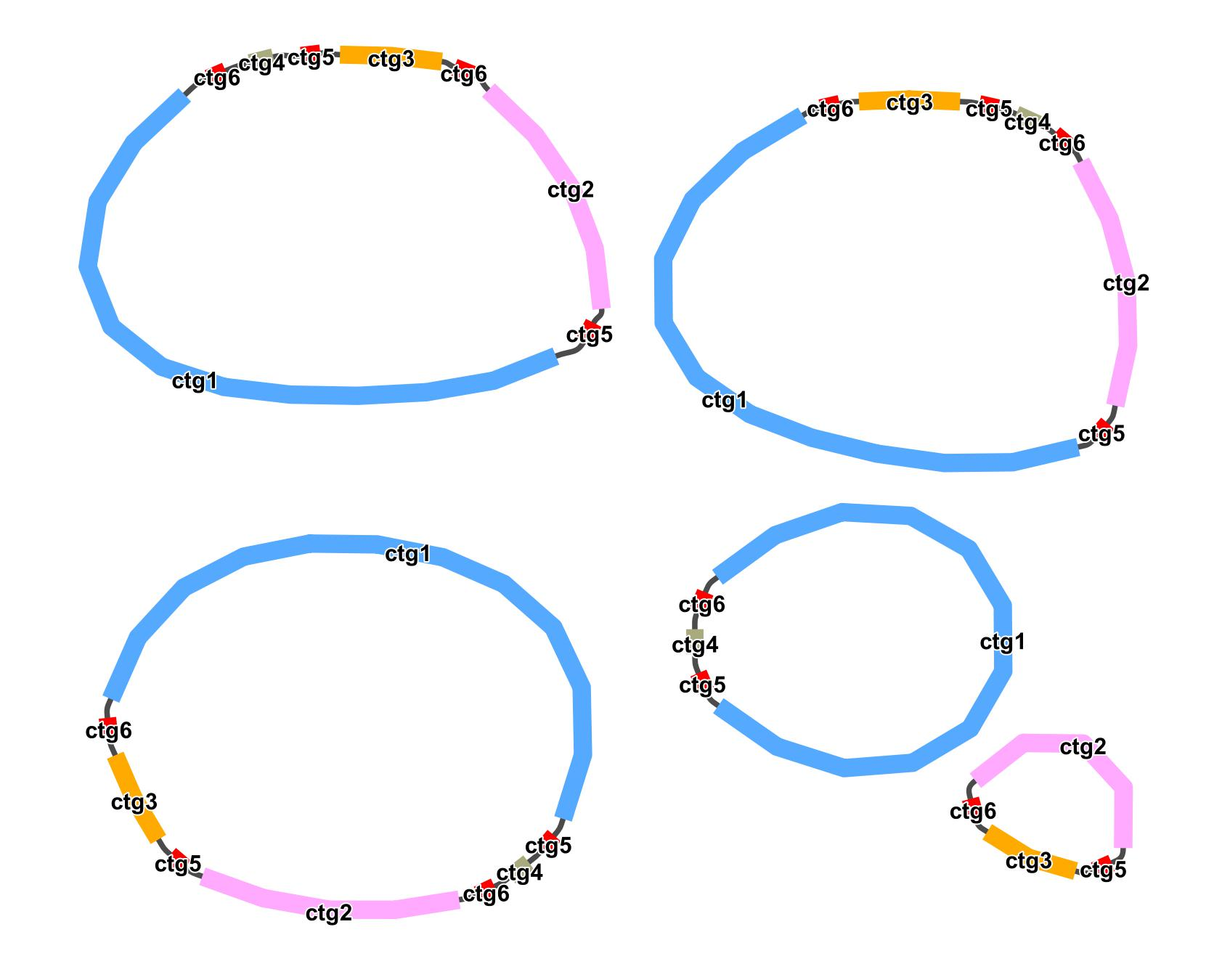


**Figure S1.** Predicted recombination configurations of the *T. amurensis* mitochondrial genome.

## Supplementary Tables

**Table S1.** Information on 35 plant species used for comparative analysis of mitochondrial whole genome.

| **Number** | **Plant name** | **Login number** |
| --- | --- | --- |
| 1 | Tilia amurensis | PQ_072837 |
| 2 | Gossypium hirsutum | NC_027406.1 |
| 3 | Gossypium barbadense | NC_028254.1 |
| 4 | Gossypium harknessii | NC_027407.1 |
| 5 | Gossypium raimondii | NC_029998.1 |
| 6 | Corchorus olitorius | NC_031360.1 |
| 7 | Corchorus capsularis | NC_031359.1 |
| 8 | Abelmoschus esculentus | OL348387_8.1 |
| 9 | Theobroma grandiflorum | NC_066895.1 |
| 10 | Theobroma cacao | NC_066894.1 |
| 11 | Aquilaria sinensis | NC_054354.1 |
| 12 | Bombax ceiba | NC_038052.1 |
| 13 | Gossypium thurberi | NC_035074.1 |
| 14 | Hibiscus cannabinus | NC_035549.1 |
| 15 | Gossypium arboreum | NC_035073.1 |
| 16 | Gossypium davidsonii | NC_035075.1 |
| 17 | Gossypium trilobum | NC_035076.1 |
| 18 | Arabidopsis lyrata | NC_081483.1 |
| 19 | Brassica napus | NC_008285.1 |
| 20 | Brassica rapa | NC_016125.1 |
| 21 | Brassica juncea | NC_016123.1 |
| 22 | Brassica oleracea | NC_016118.1 |
| 23 | Boechera stricta | NC_042143.1 |
| 24 | Arabidopsis thaliana | NC_037304.1 |
| 25 | Arabis alpina | NC_037070.1 |
| 26 | Capsella bursa-pastoris | MN746809.2 |
| 27 | Capsella rubella | NC_042883.1 |
| 28 | Sinapis arvensis | NC_031896.1 |
| 29 | Schrenkiella parvula | KT988071.2 |
| 30 | Eruca vesicaria | KF442616.1 |
| 31 | Raphanus sativus | NC_018551.1 |
| 32 | Carica papaya | NC_012116.1 |
| 33 | Batis maritima | NC_024429.1 |
| 34 | Toona ciliata | NC_065060.1 |
| 35 | Citrus unshiu | NC_057142.1 |

**Table S2.** Length and sequencing depth of each node in the *Tilia amurensis* mitochondrial genome.

| **Contig/Node** | **Length (bp)** | **Depth (×)** |
| --- | --- | --- |
| 1 | 538,262 | 37 |
| 2 | 174,620 | 38 |
| 3 | 67,678 | 34 |
| 4 | 17,028 | 29 |
| 5 | 10,787 | 59 |
| 6 | 5,463 | 60 |

**Table S3.** Annotated coding genes in the *T. amurensis* mitochondrial genome.

| **Group of genes** | **Name of genes** |
| --- | --- |
| ATP synthase | *atp*1，*atp*4，*atp*6，*atp*8，*atp*9 |
| NADH dehydrogenase | *nad*1，*nad*2，*nad*3，*nad*4，*nad*4L，*nad*5，*nad*6，*nad*7，*nad*9 |
| Cytochrome b | *cob* |
| Cytochrome c biogenesis | *ccm*B，*ccm*C，*ccm*FC，*ccm*FN |
| Cytochrome c oxidase | *cox*1，*cox*2，*cox*3 |
| Maturases | *mat*R |
| Protein transport subunit | *mtt*B |
| Ribosomal protein large subunit | *rpl*2，*rpl*5，*rpl*10，*rpl*16 |
| Ribosomal protein small subunit | *rps*1，*rps*3，*rps*4，*rps*7，*rps*10，*rps*12，*rps*13，*rps*14 |
| Succinate dehydrogenase | *sdh*3，*sdh*4 |
| Ribosome RNA | *rrn*5，*rrn*18，*rrn*26 |
| Transfer RNA | *trn*C-GCA（×2），*trn*D-GUC，*trn*E-UUC，*trn*F-GAA，*trnf*M-CAU（×2），*trn*G-GCC，*trn*HGUG，*trn*I-CAU，*trn*K-UUU，*trn*M-CAU，*trn*N-GUU（×2），*trn*P-UGG，*trn*Q-UUG，*trn*S-GCU，*trn*S-GGA（×2），*trn*S-UGA，*trn*T-CGU，*trn*T-GGU，*trn*W-CCA（×2），*trn*Y-GUA |

Note: The numbers in parentheses represent the copy number of the gene. For example, (×2) indicates that there are two copies.

**Table S4.** Relative synonymous codon usage values for each amino acid in the *T. amurensis* mitochondrial genome.

| **Amino** | **Codon 1** | **Codon 2** | **Codon 3** | **Codon 4** | **Codon 5** | **Codon 6** |
| --- | --- | --- | --- | --- | --- | --- |
|  | **RSCU** | **RSCU** | **RSCU** | **RSCU** | **RSCU** | **RSCU** |
| Ala | GCU | GCA | GCC | GCG |  |  |
|  | 1.6 | 0.97 | 0.93 | 0.49 |  |  |
| Arg | AGA | CGA | CGU | AGG | CGG | CGC |
|  | 1.46 | 1.31 | 1.21 | 0.76 | 0.7 | 0.56 |
| Asn | AAU | AAC |  |  |  |  |
|  | 1.31 | 0.69 |  |  |  |  |
| Asp | GAU | GAC |  |  |  |  |
|  | 1.4 | 0.6 |  |  |  |  |
| Cys | UGU | UGC |  |  |  |  |
|  | 1.22 | 0.78 |  |  |  |  |
| End | UAA | UGA | UAG |  |  |  |
|  | 1.5 | 0.97 | 0.53 |  |  |  |
| Gln | CAA | CAG |  |  |  |  |
|  | 1.52 | 0.48 |  |  |  |  |
| Glu | GAA | GAG |  |  |  |  |
|  | 1.38 | 0.62 |  |  |  |  |
| Gly | GGA | GGU | GGG | GGC |  |  |
|  | 1.43 | 1.34 | 0.71 | 0.52 |  |  |
| His | CAU | CAC |  |  |  |  |
|  | 1.54 | 0.46 |  |  |  |  |
| Ile | AUU | AUA | AUC |  |  |  |
|  | 1.32 | 0.86 | 0.82 |  |  |  |
| Leu | UUA | CUU | UUG | CUA | CUC | CUG |
|  | 1.5 | 1.27 | 1.25 | 0.86 | 0.58 | 0.54 |
| Lys | AAA | AAG |  |  |  |  |
|  | 1.22 | 0.78 |  |  |  |  |
| Met | AUG |  |  |  |  |  |
|  | 1 |  |  |  |  |  |
| Phe | UUU | UUC |  |  |  |  |
|  | 1.13 | 0.87 |  |  |  |  |
| Pro | CCU | CCA | CCC | CCG |  |  |
|  | 1.41 | 1.11 | 0.82 | 0.67 |  |  |
| Ser | UCU | UCA | AGU | UCC | UCG | AGC |
|  | 1.35 | 1.09 | 1.04 | 0.98 | 0.88 | 0.66 |
| Thr | ACU | ACA | ACC | ACG |  |  |
|  | 1.36 | 1.05 | 0.94 | 0.64 |  |  |
| Trp | UGG |  |  |  |  |  |
|  | 1 |  |  |  |  |  |
| Tyr | UAU | UAC |  |  |  |  |
|  | 1.53 | 0.47 |  |  |  |  |
| Val | GUU | GUA | GUG | GUC |  |  |
|  | 1.2 | 1.19 | 0.86 | 0.75 |  |  |

**Table S5.** Homologous DNA fragments in the *T. amurensis* mitochondrial genome.

| **Number** | **Length**  **(bp)** | **Identity%** | **Mismatches** | **Gap Openings** | **cp start** | **cpend** | **mt start** | **mt end** | **Gene** |
| --- | --- | --- | --- | --- | --- | --- | --- | --- | --- |
| MTPT1 | 30 | 100 | 0 | 0 | 8411 | 8440 | 79689 | 79660 | partial *trn*S-GCU |
| MTPT2 | 30 | 100 | 0 | 0 | 8411 | 8440 | 469945 | 469916 | partial *trn*S-GCU |
| MTPT3 | 521 | 99.808 | 1 | 0 | 111007 | 111527 | 830088 | 829568 | partial *trn*A-UGC |
|  | 521 | 99.808 | 1 | 0 | 142313 | 142833 | 829568 | 830088 | partial *trn*A-UGC |
| MTPT4 | 1887 | 99.417 | 11 | 0 | 21457 | 23343 | 661480 | 663366 | partial *rpo*C2;partial *rpo*C1 |
| MTPT5 | 1290 | 98.062 | 18 | 1 | 60689 | 61978 | 427908 | 426626 | partial *rbc*L |
| MTPT6 | 92 | 97.826 | 2 | 0 | 92856 | 92947 | 592327 | 592236 | partial *rpl*23 |
|  | 92 | 97.826 | 2 | 0 | 160893 | 160984 | 592236 | 592327 | partial *rpl*23 |
| MTPT7 | 80 | 97.5 | 2 | 0 | 33 | 112 | 737069 | 737148 | complete *trn*H-GUG |
| MTPT8 | 1549 | 97.03 | 27 | 5 | 58244 | 59791 | 429421 | 427891 | partial *atp*B |
| MTPT9 | 72 | 95.833 | 3 | 0 | 116282 | 116353 | 76932 | 76861 | partial *trn*N-GUU |
|  | 72 | 95.833 | 3 | 0 | 137487 | 137558 | 76861 | 76932 | partial *trn*N-GUU |
| MTPT10 | 71 | 95.775 | 3 | 0 | 78448 | 78518 | 516968 | 516898 | IGS(*clp*P-*clp*P) |
| MTPT11 | 109 | 95.413 | 4 | 1 | 48773 | 48881 | 79588 | 79695 | complete *trn*S-GGA |
| MTPT12 | 84 | 95.238 | 3 | 1 | 116280 | 116362 | 120053 | 119970 | complete *trn*N-GUU |
|  | 84 | 95.238 | 3 | 1 | 137478 | 137560 | 119970 | 120053 | complete *trn*N-GUU |
| MTPT13 | 86 | 94.186 | 4 | 1 | 7170 | 7254 | 595581 | 595496 | partial *trn*Q-UUG |
| MTPT14 | 79 | 93.671 | 5 | 0 | 57524 | 57602 | 783455 | 783533 | complete *trn*M-CAU |
| MTPT15 | 46 | 93.478 | 3 | 0 | 142963 | 143008 | 208078 | 208033 | partial *trn*I-GAU |
|  | 46 | 93.478 | 3 | 0 | 110832 | 110877 | 208033 | 208078 | partial *trn*I-GAU |
| MTPT16 | 152 | 90.132 | 9 | 4 | 37200 | 37346 | 560326 | 560176 | partial *psb*C |
| MTPT17 | 105 | 87.619 | 13 | 0 | 35758 | 35862 | 411402 | 411506 | partial *psb*D |
| MTPT18 | 191 | 85.864 | 14 | 9 | 48737 | 48914 | 469800 | 469990 | complete *trn*S-GGA |
| MTPT19 | 804 | 83.831 | 79 | 26 | 70286 | 71052 | 553260 | 552471 | partial *psb*J;complete *psb*L;complete *psb*F;partial *psb*E |
| MTPT20 | 169 | 83.432 | 21 | 3 | 32037 | 32205 | 315987 | 316148 | complete *trn*D-GUC |
| MTPT21 | 87 | 81.609 | 4 | 5 | 160582 | 160656 | 793983 | 793897 | partial *trn*I-CAU |
|  | 87 | 81.609 | 4 | 5 | 93184 | 93258 | 793897 | 793983 | complete *trn*I-CAU |
| MTPT22 | 604 | 76.159 | 73 | 37 | 72214 | 72813 | 401169 | 400633 | partial *pet*G;complete *trn*W-CCA;complete *trn*P-UGG |
